# Detection of Endosymbiont *Candidatus* Midichloria mitochondrii and Tickborne Pathogens in Humans Exposed to Tick Bites, Italy

**DOI:** 10.3201/eid2809.220329

**Published:** 2022-09

**Authors:** Giovanni Sgroi, Roberta Iatta, Piero Lovreglio, Angela Stufano, Younes Laidoudi, Jairo Alfonso Mendoza-Roldan, Marcos Antonio Bezerra-Santos, Vincenzo Veneziano, Francesco Di Gennaro, Annalisa Saracino, Maria Chironna, Claudio Bandi, Domenico Otranto

**Affiliations:** University of Bari Aldo Moro, Bari, Italy (G. Sgroi, R. Iatta, P. Lovreglio, A. Stufano, Y. Laidoudi, J.A. Mendoza-Roldan, M.A. Bezerra-Santos, F. Di Gennaro, A. Saracino, M. Chironna, D. Otranto);; University of Naples Federico II, Naples, Italy (V. Veneziano);; Osservatorio Faunistico Venatorio, Naples (V. Veneziano);; University of Milan Statale, Milan, Italy (C. Bandi);; Bu-Ali Sina University, Hamedan, Iran (D. Otranto)

**Keywords:** *Candidatus* Midichloria mitochondrii, bacteria, vector-borne infections, zoonoses, parasites, endosymbiont, Borrelia, Rickettsiae, Coxiella, One Health, Italy, ticks

## Abstract

During 2021, we collected blood and serum samples from 135 persons exposed to tick bites in southern Italy. We serologically and molecularly screened for zoonotic tickborne pathogens and only molecularly screened for *Candidatus* Midichloria mitochondrii. Overall, 62 (45.9%) persons tested positive for tickborne pathogens. *Coxiella burnetii* was detected most frequently (27.4%), along with *Rickettsia* spp. (21.5%) and *Borrelia* spp. (10.4%). We detected *Candidatus* M. mitochondrii DNA in 46 (34.1%) participants who had statistically significant associations to tickborne pathogens (p<0.0001). Phylogenetic analysis of *Candidatus* M. mitochondrii sequences revealed 5 clades and 8 human sequence types that correlated with vertebrates, *Ixodes* spp. ticks, and countries in Europe. These data demonstrated a high circulation of tickborne pathogens and *Candidatus* M. mitochondrii DNA in persons participating in outdoor activities in southern Italy. Our study shows how coordinated surveillance among patients, clinicians, and veterinarians could inform a One Health approach for monitoring and controlling the circulation of tickborne pathogens.

Hard ticks (Acarina: Ixodidae) represent a major public health issue worldwide and are vectors of a broad range of viruses, bacteria, and parasites of human and veterinary concern (*1*). Tickborne diseases are increasing globally (*2*), and several zoonotic tickborne pathogens, such as *Coxiella burnetii*, *Rickettsia* spp., and *Borrelia burgdorferi* sensu lato complex, are emerging or re-emerging in animals and humans (*3*,*4*). 

Wild animals are increasingly becoming synanthropic, and urbanization of wildlife could bring them into contact with humans, as recently observed during the COVID-19 lockdown in Chile, when sightings of several animal species not previously seen in cities were reported in urban settlements (*5*). Wildlife movement into areas of human habitation could contribute to higher circulation of ticks, tickborne pathogens, and other zoonotic agents in suburban and rural areas (*6*). However, because of the scant notifications of tickborne diseases, limited data are available on ticks and tickborne pathogens circulating in human populations (*7*). 

Because Italy is highly suited to outdoor activities such as camping, gardening, hiking, farming, and forestry work, the risk for tick bites among humans is likely high in suburban and rural environments, especially in southern regions where several species of *Ixodes* ticks have been recorded (*7*,*8*). Indeed, high seroprevalences for *Rickettsia* spp. (8.0%) and *B. burgdorferi* s.l. complex (7.5%) have been reported in forestry workers, farmers, and livestock breeders in the central-southern regions of Italy (*9*). Studies focused on humans exposed to tick bites in southern Italy also reported high seroprevalence for *C. burnetii* (16.0%) and spotted fever group rickettsiae (4.8%) that paralleled molecular detection of the same pathogens in ticks collected from the environment and infested reptiles (*4*). Those data emphasize the importance of wild animals as reservoirs of tickborne pathogens. Among categories of persons at risk for tickborne pathogen infections, hunters are more likely to be infected than hikers or forestry workers (*10*); high seroprevalence for *Rickettsia* spp. was noted in hunters from Germany (9.1%) (*11*) and Brazil (14.7%) (*12*). Also, hunting dogs are considered sentinels and reservoirs of several infectious and parasitic agents, including ticks and tickborne pathogens that can be transmitted to animals and humans (*8*,*13*). Although several epidemiologic studies have reported circulation of tickborne pathogens in animals and humans worldwide, limited data are available on tick endosymbionts and their role in the transmission of pathogens or clinical onset of tickborne diseases in vertebrate hosts.

For instance, *Candidatus* Midichloria mitochondrii is an intracellular bacterial endosymbiont of ticks, which is detected inside mitochondria and has high prevalence in the sheep tick, *Ixodes ricinus* (*14*). This bacterium is vertically transmitted through generations of *I. ricinus* ticks, but strong evidence supports its transmission to vertebrate hosts via a blood meal, which has been experimentally reported in rabbits (*15*). Indeed, high *Candidatus* M. mitochondrii seroprevalence (58%) has been reported in human patients exposed to *I. ricinus* tick bites (*16*) and those affected by Lyme disease (*17*). Although these results support the idea that *Candidatus* M. mitochondrii is inoculated into the vertebrate host during the blood meal of the tick, whether this bacterium is involved in tickborne pathogen transmission remains unclear. However, screening studies for *Candidatus* M. mitochondrii DNA in human blood samples are lacking, even though DNA has been detected in several anthropophilic tick species, such as *Rhipicephalus turanicus*, *Rhipicephalus bursa*, and *Haemaphysalis punctata* (*18*); in domestic dogs, sheep, and horses (*19*); in wild roe deer (*Capreolus capreolus*) (*20*); and in laboratory rabbits (*15*).

We aimed to assess the occurrence of zoonotic tickborne pathogens in persons exposed to tick bites and living in suburban and rural areas of southern Italy. In addition, we investigated the presence and the potential involvement of *Candidatus* M. mitochondrii in tickborne pathogen infections.

## Methods

### Study Area

The study was conducted in 3 regions of southern Italy, Apulia, Basilicata, and Campania, which are surrounded by the Adriatic, Ionian, and Tyrrhenian Seas. These regions are characterized by a typical Mediterranean temperate climate and have progressively continental features including inland and mountainous landscapes.

### Sampling

During February–December 2021, we collected blood and serum samples from 135 persons exposed to tick bites by using 10 mL BD Vacutainer EDTA Blood Collection Tubes and 5 mL BD Vacutainer Serum-separating Tubes (Beckton Dickenson, https://www.bd.com). For each participant, we used a detailed anamnestic form to collect data on patient age, sex, participation in outdoor activities (i.e., hiking, hunting, camping, gardening, farming, or forestry work), geographic origin of tick bite, and post-bite clinical findings ascribable to tickborne diseases. All samples were kept at +4°C in portable refrigerators and delivered to the Departments of Biomedical Science and Human Oncology and Veterinary Medicine, University of Bari (Bari, Italy), for serologic and molecular analyses. 

This study was conducted in accordance with ethical principles of the Declaration of Helsinki. Patients provided written informed consent after they were fully informed about the research aims and features. The research was approved by the ethics committee of the University Hospital of Bari Aldo Moro (Italy) (approval no. 6394, protocol no. 0044469-23062020).

### Serologic Assays

We screened all serum samples for pathogen-specific IgG by using indirect chemiluminescent immunoassays, *Coxiella burnetii* VirCLIA IgG, *Rickettsia* *conorii* VirCLIA IgG, and *Borrelia* VirCLIA IgG (Vircell, https://en.vircell.com). Results were expressed by antibody concentration index, which we calculated as the ratio between the sample and calibrator relative light units and interpreted according to the manufacturer’s instructions.

The *C. burnetii* IgG test showed a sensitivity of 90%, specificity of 100%, positive predictive value of 100%, and negative predictive value of 96%. The *R. conorii* IgG test showed sensitivity of 100%, specificity of 100%, positive predictive value of 100%, and negative predictive value of 100%. The *Borrelia* IgG test showed sensitivity of 89%, specificity of 98%, positive predictive value of 97%, and negative predictive value of 92%.

### DNA Extraction, PCR, and Phylogenetic Analysis

We used the QIAampDNA Blood & Tissue Kit (QIAGEN, https://www.qiagen.com) to extract DNA from blood samples, according to the manufacturer’s instructions, and tested for tickborne pathogens and *Candidatus* M. mitochondrii. In all PCR runs, we used sterile water as the negative control and used the following as positive controls: *Anaplasma phagocytophilum* and *Candidatus* M. mitochondrii for PCR targeting 16S rRNA gene; *C. burnetii* for IS1111a and *Borrelia lusitaniae* for Flagellin from *I. ricinus* ticks; and *Rickettsia raoultii* for *gltA* from a *Dermacentor marginatus* tick specimen (Table 1) (*21*–*24*). All ticks used were previously collected from animals or humans in the same study area. 

**Table 1:** Bacteria investigated by molecular methods in a study on the relationship between endosymbiont *Candidatus* Midichloria mitochondrii and tickborne pathogens in humans exposed to tick bites, Italy, 2021

Tickborne bacteria	Target gene	Primers	Sequence, 5ˈ → 3ˈ	Base pairs	Reference
*Coxiella burnetii*	IS1111a	Trans1	TATGTATCCACCGTAGCCAGT	687	(*21*)
		Trans2	CCCAACAACACCTCCTTATTC		
*Rickettsia* spp.	*gltA*	CS 78F	GCAAGTATCGGTGAGGATGTAAT	401	(*22*)
		CS 323R	GCTTCCTTAAAATTCAATAAATCAGGAT		
*Borrelia burgdorferi* sensu lato complex	Flagellin	FLA1	AGAGCAACTTACAGACGAAATTAAT	482	(*23*)
		FLA2	CAAGTCTATTTTGGAAAGCACCTAA		
*Anaplasmataceae*	16S rRNA	EHR 16SD	GGTACCYACAGAAGAAGTCC	345	(*24*)
		EHR 16SR	TAGCACTCATCGTTTACAGC		

We placed PCR products on 2% agarose gel stained with GelRed Nucleic Acid Gel Stain (Biotium-VWR International, https://us.vwr.com) and visualized products on a Gel Logic 100 Digital Imaging System (Kodak, https://www.kodak.com). We then purified amplicons and sequenced in both directions by using the same primers we used for PCR and by using the BigDye Terminator v3.1 Cycle Sequencing Kit (Thermo Fisher Scientific, https://www.thermofisher.com) in a 3130 Genetic Analyzer (Applied Biosystems/Thermo Fisher Scientific). We edited and analyzed sequences by using Geneious version 9.0 (https://www.geneious.com) and compared the obtained sequences with available sequences in the GenBank database by using BLAST (http://blast.ncbi.nlm.nih.gov/Blast.cgi).

We used MAFFT version 7.490 (*25*) to align the 16S rRNA gene sequences obtained from human blood samples against 123 available *Midichloria* genus entries in GenBank, including a sequence type from *I. ricinus* (accession no. MZ954838) previously found in the study area (*8*). We then trimmed sequences by using TrimAL version 1.4 revision 15 (*26*). We split the multisequence alignment (MSA) file into 2 partitions: a reference sequence alignment (RefMSA) containing 1 reference sequence for each sequence type (38 sequences with 202 nucleotide sites) and a query sequence alignment containing the remaining 115 sequences. We created a maximum-likelihood phylogeny on the RefMSA by using IQ-TREE multicore version 1.6.12 (*27*) under 1,000 ultrafast bootstrap replications (*28*). We selected the Kimura 3-parameter model with empirical frequencies plus regression (TPM3+F+R2) model in ModelFinder (*29*), which we implemented as function in the IQ-TREE computation. We used the DNA sequence of *Lariskella* endosymbiont of *Curculio okumai* beetles (GenBank accession no. AB746416) as an outgroup. To assess the species diversity among the genus *Midichloria*, we performed a multi-rate Poisson tree processes on the maximum-likelihood tree by using mPTP version 0.2.4_osx_x86_64 (*30*). We used an accurate phylogenetic placement based on the least squares method (*31*) to place the query sequences on the maximum-likelihood tree. In brief, we subjected the maximum-likelihood tree generated by IQ-TREE to branch length correction using FastTree 2 software (http://www.microbesonline.org/fasttree). We then used the corrected tree backbone, the RefMSA, and query sequence alignments to generate the phylogenetic placement by using APPLES version 2.0.6 (*31*). Finally, we used the result of species delimitation and informative alignment to annotate the final tree by using iTOL version 5 (*32*). We performed Pearson correlation to assess the relationship between phylogenetic clades of *Midichloria* sequences and their host or isolation source and geographic origin. We performed all analyses on RStudio software (*33*).

### Statistical Analysis

We established exact binomial 95% CI for the prevalence values found. We used a χ*^2^* test to assess statistical differences of prevalence of tickborne pathogens with age, sex, outdoor activity, and geographic origin of tick bite of the enrolled patients. We considered p<0.05 statistically significant. We calculated the odds ratio (OR) to assess the infection risk according to several participant variables. We used a κ value formula to compare serologic and molecular methods and considered risk agreement poor at 0–20%, fair at 21%–40%, moderate at 41%–60%, strong at 61%–80%, and perfect at 81%–100%.

We used Epitools Epidemiologic Calculators software (*34*) to calculate 95% CI, χ^2^, p value, OR, and κ values. We used QGIS3 version 3.10.2 with GRASS 7.8.2 (QGIS Project, https://www.qgis.org) to map the distribution of participants who were positive for tickborne pathogens and *Candidatus* M. mitochondrii in the administrative boundaries of the regional study area.

## Results

Among 135 participants, 47.4% (64/135) reported having been bitten by ticks in the last year, and 62 (45.9%, 95% CI 37.7%–54.3%) tested positive for >1 tickborne pathogen, 54 (40.0%, 95% CI 32.1%–48.4%) by serologic methods and 20 (14.8%, 95% CI 9.8%–21.8%) by molecular methods. We identified *C. burnetii* from 37 (27.4%) participants, *Rickettsia* spp. from 29 (21.5%), and *Borrelia* spp. from 14 (10.4%). We serologically detected tickborne pathogen co-infections in 14 (10.4%) participants, most of whom had *C. burnetii*–*Rickettsia* spp. coinfection. We detected co-infection by molecular methods in 1 (0.7%) case-patient who had *R. raoultii–B. lusitaniae* coinfection.

Nine persons were positive for the same pathogen by both diagnostic tools; 4 were positive for *C. burnetii*, 4 for *R. raoultii*, and 1 for *B. lusitaniae*. We noted strong agreement between serologic and molecular results (κ = 63.0%) (Table 2).

**Table 2:** Serologic and PCR findings for tickborne pathogens in humans exposed to tick bites, Italy, 2021*

Tickborne pathogens	Serology			PCR	
	No. patients	% Patients (95% CI)†		No. patients (%)	% Patients (95% CI)†
Single infections					
* Coxiella burnetii*	21	15.6 (10.4–22.6)		7	5.2 (2.5–10.3)
* Rickettsia raoultii*	14	10.4 (6.3–16.7)		6	4.4 (2.0–9.4)
* Borrelia lusitaniae*	5	5 (1.6–8.4)		6	4.4 (2.0–9.4)
Co-infections					
*Coxiella burnetii*, *Rickettsia* spp.	11	11 (4.6–14.0)		NA	NA
*Coxiella burnetii*, *Borrelia* spp.	2	2 (0.4–5.2)		NA	NA
*Rickettsia raoultii*, *Borrelia lusitaniae*	1	1 (0.1–4.1)		1	0.7 (0.1–4.1)
Total	54	54 (32.1–48.4)		20	14.8 (9.8–21.8)

*Based on 135 persons tested. NA, not applicable †Exact binomial 95% CI.

Among the 62 participants who tested positive for tickborne pathogens, 31 (50.0%) reported a localized reaction (i.e., edema and redness) at the tick bite site, among whom 13 (20.6%) displayed >1 clinical sign ascribable to tickborne pathogen infections (Table 3). We noted a statistically significant association between the occurrence of tickborne pathogens and administrative region among patients living in Basilicata, suggesting this area could have higher levels of tickborne pathogens (Table 4).

**Table 3:** Clinical findings in 13 patients tested positive for tickborne pathogens, Italy, 2021*

Patient no.	Tickborne pathogens detected	Clinical findings				
		Rash	Axillary lymphadenomegaly	Fever	Myalgia	Headache
1	Rickettsia raoultii*	N	Y	N	N	N
2	*Rickettsia* spp.†	N	N	N	N	Y
3	*Coxiella burnetii*†	N	Y	Y	Y	N
4	Borrelia lusitaniae*	N	N	Y	Y	N
5	*Borrelia* spp.†	N	N	Y	Y	N
6	*Rickettsia* spp.†	N	Y	Y	Y	N
7	*Rickettsia* spp.†	N	N	Y	N	N
8	B. lusitaniae*	N	N	Y	Y	N
9	*Rickettsia* spp.†	N	N	Y	Y	Y
10	*Rickettsia* spp.†	N	N	Y	Y	N
11	*C. burnetii*†	N	Y	N	N	N
12	*Borrelia* spp.†, *Rickettsia *spp.†, B. lusitaniae*	Y‡	N	Y	N	N
13	R. raoultii*	Y§	N	N	N	N


*Pathogen detected by PCR. †Pathogen detected by serology. ‡Maculopapular rash. §Erythematous macular rash.

**Table 4:** Characteristics of persons tested by serology and PCR for tickborne pathogens in humans exposed to tick bites, Italy, 2021*

Characteristics	Serology†					PCR†			
	No. positive/no. tested	% Positive (95% CI)	p value	Χ^2^		No. positive/no. tested	% Positive (95% CI)	p value	Χ^2^
Age range, y			0.396	0.7				0.614	0.2
25–49	22/61	36.1 (25.2–48.6)				8/61	13.1 (6.8–23.8)		
50–68	32/74	43.2 (32.6–54.6)				12/74	16.2 (9.5–26.2)		
Sex			0.396	0.7				0.120	2.4

M	43/112	38.4 (29.9–47.6)				19/112	17.0 (11.1–25.0)		
F	11/23	47.8 (29.2–67.0)				1/23	4.3 (0.7–21.0)		
Outdoor activity			0.990	0.1				0.190	4.7
Farming	7/18	38.4 (20.3–61.4)				1/18	5.6 (1.0–25.7)		
Hunting	26/65	40.0 (29.0–52.1)				9/65	13.8 (7.5–24.3)		
Camping, gardening	12/31	38.7 (23.7–56.2)				8/31	25.8 (13.7–3.2)		

Hiking, forestry work	9/21	42.9 (24.5–63.4)				2/21	9.5 (2.6–28.9)		
Administrative region			0.030	6.9				0.002	12.3
Apulia	8/36	22.2 (11.7–38.1)				2/36	5.6 (1.5–18.1)		
Basilicata	17/33	51.5 (35.2–67.5)				11/33	33.3 (19.7–50.4)		
Campania	29/66	43.9 (32.6–55.9)				7/66	10.6 (5.2–20.3)		
Total	54/135	40.0 (32.1–48.4)				20/135	14.8 (9.8–21.8)		

*Number and percentage of patients testing positive out of the total number examined for each category. †Exact binomial 95% CI is given; Χ^2^ and p values are given for each category.

**Figure 1:**
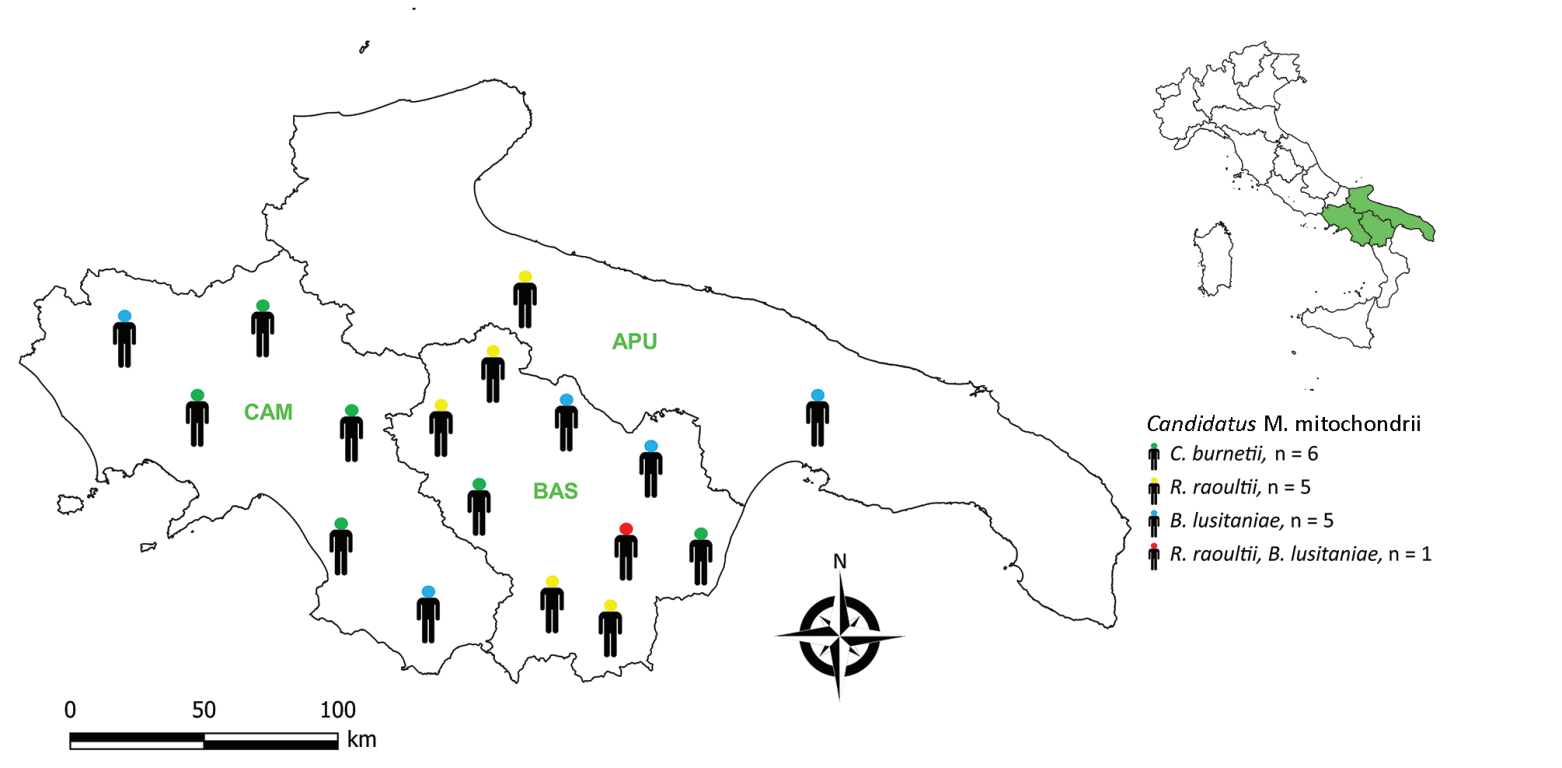
Distribution of 17 persons in whom *Candidatus* Midichloria mitochondrii and tickborne pathogens were detected, Italy, 2021. Inset shows region of interest in southern Italy (green shading). APU, Apulia; BAS, Basilicata; *B. lusitaniae*, *Borrelia lusitaniae*; CAM, Campania; *C. burnetii*, *Coxiella burnetii*; *R. raoultii*, *Rickettsia raoultii*.

We detected *Candidatus* M. mitochondrii DNA in 46 (34.1%, 95% CI 26.6%–42.4%) participants, among whom 11 tested negative for tickborne pathogens and 35 tested positive for >1 tickborne pathogen. The 35 participants who tested positive for tickborne infections and *Candidatus* M. mitochondrii DNA represented 18/54 (33.3%, 95% CI 22.2%–46.6%) of participants whose infections were detected serologically and 17/20 (85.0%, 95% CI 63.9%–94.7%) of participants whose infections were detected molecularly. *Candidatus* M. mitochondrii was statistically associated with each pathogen found (i.e., *C. burnetii*, *R. raoultii*, and *B. lusitaniae*) (Table 5; Figure 1). No clinical signs were reported in persons infected by *Candidatus* M. mitochondrii and we saw no statistically significant difference in the clinical signs between subjects who tested positive for tickborne pathogens and those who were positive for both tickborne pathogens and *Candidatus* M. mitochondrii (χ*^2^* = 0.9, p = 0.342). A single case of *Candidatus* Wolbachia inokumae was molecularly detected in a participant infected by *B. lusitaniae*.

**Table 5:** Tickborne pathogens and *Candidatus* Midichloria mitochondrii detected in humans exposed to tick bites, Italy, 2021*

Tickborne pathogens	No. patients	*Candidatus* M. mitochondrii, no.†	% Patients (95% CI)‡	Χ^2^	p value	OR
*Coxiella burnetii*	7	6	85.7 (48.7–97.4)	8.8	0.003	13.2
*Rickettsia raoultii*	6	5	83.3 (43.6–97.0)	6.9	0.008	11.0
*Borrelia lusitaniae*	6	5	83.3 (43.6–97.0)	6.9	0.008	11.0
*R. raoultii–B. lusitaniae*	1	1	NA	NA	NA	NA
Total	20	17	85.0 (63.9–94.7)	26.0	.001	16.1

*NA, not applicable; OR, odds ratio. †Number and percentage of patients positive for tickborne pathogens and *Candidatus* M. mitochondrii. ‡Exact binomial 95% CI.

Consensus sequences of all bacteria species we detected displayed 99%–100% nucleotide identity with sequences available in the literature. We submitted sequences to GenBank under the following accession numbers: ON227500 for *C. burnetii*, ON228179 for *R. raoultii*, ON237925 for *B. lusitaniae*, OM982495–2502 for *M. mitochondrii*, and OM983334 for *W. inokumae*. 

**Figure 2:**
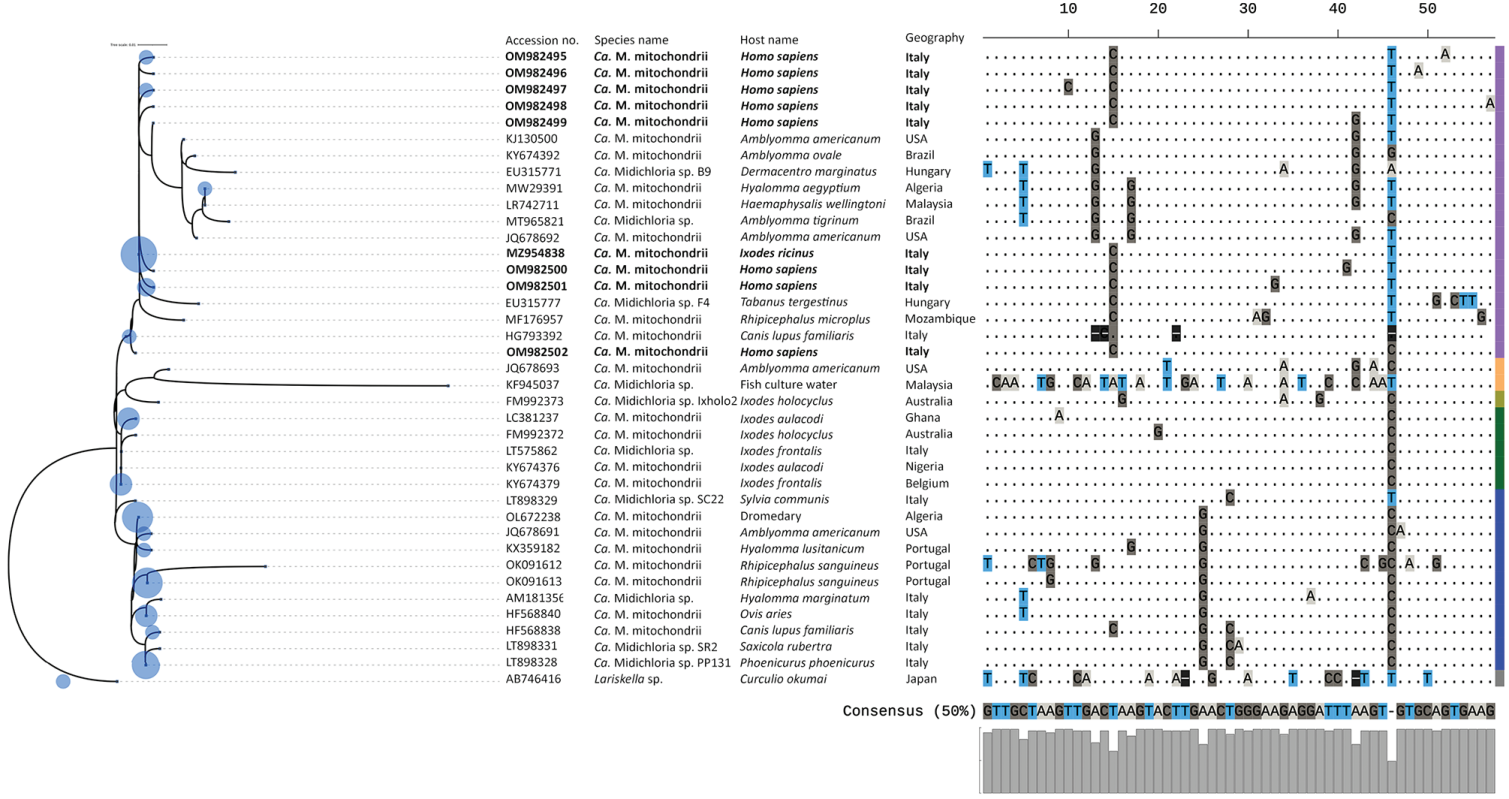
Maximum-likelihood phylogenic tree of endosymbiont *Candidatus *Midichloria mitochondrii clades detected with tickborne pathogens in humans exposed to tick bites, Italy, 2021. The tree corresponds to the IQ-TREE (http://www.iqtree.org) inferred from 38 partial (202 bp) DNA sequences with 28.7% of informative sites by using the Kimura 3-parameter model with empirical frequencies plus regression (TPM3+F+R2) model under 1,000 bootstrap replicates and maximum-likelihood method. Accession numbers and species name are indicated at the tip of each branch. Bold blue text indicates sequences amplified in the study area. The tree includes 123 query sequences representing all 16S rRNA entries from GenBank (blue circles on left) placed at the branch and leaf nodes by using the APPLES algorithm (https://github.com/balabanmetin/apples). The ClustalX (http://www.clustal.org/clustal2) sequence alignment viewer of the informative sites from the 16S rRNA alignment and their 50% consensus are shown. Colored column at far right denotes taxonomic annotation; purple indicates clade A, blue indicates clade B, green indicates clade C, yellow indicates clade D, green indicates clade E, gray indicates outgroup. *Ca*., *Candidatus*.

Phylogenetic analysis of the partial 16S rRNA gene revealed 8 sequence types and the existence of 5 distinct clades (clades A–E); the *Candidatus* M. mitochondrii sequence types we obtained belonged clade A (Figure 2). Pearson correlation (*r*) showed that *Candidatus* M. mitochondrii clade A was significantly correlated with vertebrate hosts (*r* = 0.32), *Ixodes* spp. ticks (*r* = 0.21), and countries in Europe (*r* = 0.46); clade B with *Hyalomma* ticks (*r* = 0.4) and Argasidae ticks (*Ornithodoros* spp.) (*r* = 0.36) and Africa (*r* = 0.48); clade C with *Ixodes* spp. ticks (*r* = 0.46); and clades D and E with marine sources (*r* = 0.57) (Appendix Figure).

## Discussion

We report a high serologic and molecular prevalence of infection by zoonotic tickborne bacteria in humans exposed to tick bites in rural areas of southern Italy. In addition, we noted an association between *Candidatus* M. mitochondrii and tickborne pathogens in humans, suggesting that this tick endosymbiont might be involved in these infections.

The finding of *C. burnetii* as the most representative tickborne pathogen in humans from southern Italy is a public health concern because prevalence of this bacterium in hard ticks is much higher in the Mediterranean Basin than in other areas of Europe (*35*). However, the high (27.4%) overall serologic and molecular prevalence we obtained contrasts with the low (0.5%) rate of molecular detection of the bacterium in ticks collected on citizens in the same study area (*8*). This finding suggests inhalation of contaminated aerosols, not tick bites, might be the main route of pathogen transmission among the study participants (*36*).

Detection of *R*. *raoultii* is relevant because this pathogen is considered an emerging spotted fever group rickettsiae among humans (*37*,*38*). Although no patient in our study showed the scalp eschar and neck lymphadenopathy that are typical clinical signs for differential diagnosis (*39*), the difference in clinical features might be dependent on the site of the tick bite on a person’s body (*40*).

Detection of *B. lusitaniae* in persons frequenting rural environments of southern Italy confirms the circulation of this zoonotic genospecies, which previously was reported in different hosts, such as the Italian wall lizard (*Podarcis siculus*) (*41*) and red foxes (*Vulpes vulpes*) (*42*), and in *I. ricinus* ticks collected on humans from the same study area (*8*). The human pathogenic role of *B. lusitaniae* has not been completely clarified, but the finding of erythematous macules from positive patients examined in this study is of clinical relevance because these skin lesions could cause chronic or long-lasting injuries associated with infiltration of the local subcutaneous tissues (*43*). In addition, co-infections in this human population and in ticks previously collected from citizens of the same study area (*8*), indicate that multiple pathogens could be transmitted by the same tick specimen and develop in the same host, complicating the clinical manifestation (*7*).

Despite the strong agreement (κ = 63.0%) in the comparison between serology and PCR, use of both serologic and molecular methods is crucial for identifying all cases of infection (*44*). Indeed, combining PCR and serology can be useful for detecting recent and old infections because PCR is sensitive <2 weeks of infection and serology is sensitive >2 weeks after illness onset (*17*).

The high molecular prevalence (34.1%) of *Candidatus* M. mitochondrii in participants exposed to tick bites is in accordance with the serologic rate (47.3%) previously outlined for this endosymbiont in humans (*16*), and confirms its role as a candidate tick-bite marker (*17*). Thus, because *Candidatus* M. mitochondrii is known to subsist in several hard tick species (*18*), its detection in human blood could be a useful tool for determining exposure to tick bites and to define tick populations circulating in a certain area. The statistical association between molecular positivity for tickborne pathogens and *Candidatus* M. mitochondrii demonstrated here suggests a link between endosymbiont and tickborne pathogen infections in humans, which was previously established through serologic methods (*16*). However, absence of clinical signs or symptoms in humans testing positive for *Candidatus* M. mitochondrii and the statistically significant difference in the clinical picture between participants who tested positive only for tickborne pathogens and those who tested positive for both tickborne pathogens and *Candidatus* M. mitochondrii, suggests that this bacterium might be not involved in the onset or overt pathogenesis of primary infections. Conversely, the hypothesis of a potential *Candidatus* M. mitochondrii–tickborne pathogen interaction within tick hosts needs further investigation, as previously demonstrated for this endosymbiont in regulating the growth of *R. parkeri* in its competent vector *Amblyomma maculatum* ticks (*45*).

Our results also highlight the occurrence of 8 *Candidatus* M. mitochondrii sequence types, suggesting patients were exposed to different tick species, which reinforces the hypothesis that *Candidatus* M. mitochondrii could be used as candidate marker to define the composition of the tick population biting humans in a certain area (*4*). In addition, the clustering of all sequence types into clade A, as we noted, is relevant for public health because this clade encompasses endosymbionts associated with vertebrate hosts, rather than to *Ixodes* spp. ticks, and geographic areas of Europe. On the other hand, the presence of the same *Midichloria* genotype within several tick species indicates that vertebrates might act as ecological arenas for intraspecies and interspecies–specific transmission of endosymbionts among hard ticks, which was previously suggested by similar 16S rRNA sequences in genetically distant tick species (*46*). Another phylogenetic study analyzed concatenated loci of 16S rRNA, and *groEL* and *dnaK* gene sequences, and reported 3 distinct clades (I, II, and III) of *Candidatus* M. mitochondrii associated with ticks, indicating the existence of 2 lineages including different tick species and 1 *Ixodes* genus (*47*).

Detection of *W. inokumae* DNA in humans might be a consequence of exposure to a blood meal by selected phlebotomine sandflies. In fact, *W. inokumae* has only been identified in *Phlebotomus perniciosus* sand fly specimens from France and Tunisia (*48*,*49*), where it has been indicated as endosymbiont of this arthropod species in the Mediterranean Basin.

Our data demonstrate a high circulation of tickborne pathogens and *Candidatus* M. mitochondrii in persons frequenting suburban and rural areas of southern Italy. Further experimental studies could clarify the biologic interaction between tickborne pathogens and *Candidatus* M. mitochondrii and the potential role of *Candidatus* M. mitochondrii in tickborne pathogen transmission to mammalian hosts. The set of 16S rRNA sequences and their cladogenetic classification we generated could be a useful template for future studies, especially for further description of new *Candidatus* M. mitochondrii clades. 

In conclusion, the survey we describe relies on cooperation between different stakeholders, including patients, clinicians, and veterinarians. This type of surveillance could be a small step toward a One Health approach for monitoring and controlling the circulation of tickborne pathogens in suburban and rural areas (*50*).

Appendix Additional information on detection of endosymbiont *Candidatus* Midichloria mitochondrii and tickborne pathogens in humans exposed to tick bites, Italy.

## References

[1] Dantas-Torres F, Chomel BB, Otranto D. Ticks and tick-borne diseases: a One Health perspective. Trends Parasitol. 2012;28:437–46. 10.1016/j.pt.2012.07.003 22902521

[2] Kuehn B. Tickborne diseases increasing. JAMA. 2019;321:138. 30644989 10.1001/jama.2018.20464

[3] Madison-Antenucci S, Kramer LD, Gebhardt LL, Kauffman E. Emerging tick-borne diseases. Clin Microbiol Rev. 2020;33:e00083–18. 10.1128/CMR.00083-18 31896541 PMC6941843

[4] Mendoza-Roldan JA, Ravindran Santhakumari Manoj R, Latrofa MS, Iatta R, Annoscia G, Lovreglio P, et al. Role of reptiles and associated arthropods in the epidemiology of rickettsioses: A one health paradigm. PLoS Negl Trop Dis. 2021;15:e0009090. 10.1371/journal.pntd.0009090 33596200 PMC7888606

[5] Silva-Rodríguez EA, Gálvez N, Swan GJF, Cusack JJ, Moreira-Arce D. Urban wildlife in times of COVID-19: What can we infer from novel carnivore records in urban areas? Sci Total Environ. 2021;765:142713. 10.1016/j.scitotenv.2020.142713 33077221 PMC9757141

[6] Sgroi G, Iatta R, Lia RP, D’Alessio N, Manoj RRS, Veneziano V, et al. Spotted fever group rickettsiae in *Dermacentor marginatus* from wild boars in Italy. Transbound Emerg Dis. 2021;68:2111–20. 10.1111/tbed.13859 32986912

[7] Otranto D, Dantas-Torres F, Giannelli A, Latrofa MS, Cascio A, Cazzin S, et al. Ticks infesting humans in Italy and associated pathogens. Parasit Vectors. 2014;7:328. 10.1186/1756-3305-7-328 25023709 PMC4223688

[8] Sgroi G, Iatta R, Lia RP, Napoli E, Buono F, Bezerra-Santos MA, et al. Tick exposure and risk of tick-borne pathogens infection in hunters and hunting dogs: a citizen science approach. Transbound Emerg Dis. 2022;69:e386–93. 10.1111/tbed.14314 34487635 PMC9546254

[9] Santino I, Cammarata E, Franco S, Galdiero F, Oliva B, Sessa R, et al. Multicentric study of seroprevalence of *Borrelia burgdorferi* and *Anaplasma phagocytophila* in high-risk groups in regions of central and southern Italy. Int J Immunopathol Pharmacol. 2004;17:219–23. 10.1177/039463200401700214 15171823

[10] Toepp AJ, Willardson K, Larson M, Scott BD, Johannes A, Senesac R, et al. Frequent exposure to many hunting dogs significantly increases tick exposure. Vector Borne Zoonotic Dis. 2018;18:519–23. 10.1089/vbz.2017.2238 30016206 PMC6426272

[11] Jansen A, La Scola B, Raoult D, Lierz M, Wichmann O, Stark K, et al. Antibodies against *Rickettsia* spp. in hunters, Germany. Emerg Infect Dis. 2008;14:1961–3. 10.3201/eid1412.080229 19046538 PMC2634617

[12] Kmetiuk LB, Krawczak FS, Machado FP, Paploski IAD, Martins TF, Teider-Junior PI, et al. Ticks and serosurvey of anti-*Rickettsia* spp. antibodies in wild boars (*Sus scrofa*), hunting dogs and hunters of Brazil. PLoS Negl Trop Dis. 2019;13:e0007405. 10.1371/journal.pntd.0007405 31145746 PMC6542515

[13] Sgroi G, Varcasia A, Dessì G, D’Alessio N, Pacifico L, Buono F, et al. Massive *Taenia hydatigena* cysticercosis in a wild boar (*Sus scrofa*) from Italy. Acta Parasitol. 2019;64:938–41. 10.2478/s11686-019-00110-3 31444647

[14] Stavru F, Riemer J, Jex A, Sassera D. When bacteria meet mitochondria: The strange case of the tick symbiont Midichloria mitochondrii^†^. Cell Microbiol. 2020;22:e13189. 10.1111/cmi.13189 32185904

[15] Cafiso A, Sassera D, Romeo C, Serra V, Hervet C, Bandi C, et al. *Midichloria mitochondrii*, endosymbiont of *Ixodes ricinus*: evidence for the transmission to the vertebrate host during the tick blood meal. Ticks Tick Borne Dis. 2019;10:5–12. 10.1016/j.ttbdis.2018.08.008 30154059

[16] Mariconti M, Epis S, Gaibani P, Dalla Valle C, Sassera D, Tomao P, et al. Humans parasitized by the hard tick *Ixodes ricinus* are seropositive to *Midichloria mitochondrii*: is *Midichloria* a novel pathogen, or just a marker of tick bite? Pathog Glob Health. 2012;106:391–6. 10.1179/2047773212Y.0000000050 23265610 PMC3589662

[17] Serra V, Krey V, Daschkin C, Cafiso A, Sassera D, Maxeiner HG, et al. Seropositivity to *Midichloria mitochondrii* (order Rickettsiales) as a marker to determine the exposure of humans to tick bite. Pathog Glob Health. 2019;113:167–72. 10.1080/20477724.2019.1651568 31397213 PMC6758646

[18] Epis S, Sassera D, Beninati T, Lo N, Beati L, Piesman J, et al. *Midichloria mitochondrii* is widespread in hard ticks (Ixodidae) and resides in the mitochondria of phylogenetically diverse species. Parasitology. 2008;135:485–94. 10.1017/S0031182007004052 18205982

[19] Bazzocchi C, Mariconti M, Sassera D, Rinaldi L, Martin E, Cringoli G, et al. Molecular and serological evidence for the circulation of the tick symbiont *Midichloria* (Rickettsiales: Midichloriaceae) in different mammalian species. Parasit Vectors. 2013;6:350. 10.1186/1756-3305-6-350 24330701 PMC4029736

[20] Serra V, Cafiso A, Formenti N, Verheyden H, Plantard O, Bazzocchi C, et al. Molecular and serological evidence of the presence of *Midichloria mitochondrii* in roe deer (*Capreolus capreolus*) in France. J Wildl Dis. 2018;54:597–600. 10.7589/2017-09-241 29547359

[21] Berri M, Laroucau K, Rodolakis A. The detection of *Coxiella burnetii* from ovine genital swabs, milk and fecal samples by the use of a single touchdown polymerase chain reaction. Vet Microbiol. 2000;72:285–93. 10.1016/S0378-1135(99)00178-9 10727838

[22] Labruna MB, Whitworth T, Horta MC, Bouyer DH, McBride JW, Pinter A, et al. *Rickettsia* species infecting *Amblyomma cooperi* ticks from an area in the state of São Paulo, Brazil, where Brazilian spotted fever is endemic. J Clin Microbiol. 2004;42:90–8. 10.1128/JCM.42.1.90-98.2004 14715737 PMC321730

[23] Wójcik-Fatla A, Szymańska J, Wdowiak L, Buczek A, Dutkiewicz J. Coincidence of three pathogens (*Borrelia burgdorferi* sensu lato, *Anaplasma phagocytophilum* and *Babesia microti*) in *Ixodes ricinus* ticks in the Lublin macroregion. Ann Agric Environ Med. 2009;16:151–8. 19630205

[24] Parola P, Roux V, Camicas JL, Baradji I, Brouqui P, Raoult D. Detection of *ehrlichiae* in African ticks by polymerase chain reaction. Trans R Soc Trop Med Hyg. 2000;94:707–8. 10.1016/S0035-9203(00)90243-8 11198664

[25] Nakamura T, Yamada KD, Tomii K, Katoh K. Parallelization of MAFFT for large-scale multiple sequence alignments. Bioinformatics. 2018;34:2490–2. 10.1093/bioinformatics/bty121 29506019 PMC6041967

[26] Capella-Gutiérrez S, Silla-Martínez JM, Gabaldón T. trimAl: a tool for automated alignment trimming in large-scale phylogenetic analyses. Bioinformatics. 2009;25:1972–3. 10.1093/bioinformatics/btp348 19505945 PMC2712344

[27] Minh BQ, Schmidt HA, Chernomor O, Schrempf D, Woodhams MD, von Haeseler A, et al. IQ-TREE 2: new models and efficient methods for phylogenetic inference in the genomic era. Mol Biol Evol. 2020;37:1530–4. 10.1093/molbev/msaa015 32011700 PMC7182206

[28] Hoang DT, Chernomor O, von Haeseler A, Minh BQ, Vinh LS. UFBoot2: improving the ultrafast bootstrap approximation. Mol Biol Evol. 2018;35:518–22. 10.1093/molbev/msx281 29077904 PMC5850222

[29] Kalyaanamoorthy S, Minh BQ, Wong TKF, von Haeseler A, Jermiin LS. ModelFinder: fast model selection for accurate phylogenetic estimates. Nat Methods. 2017;14:587–9. 10.1038/nmeth.4285 28481363 PMC5453245

[30] Zhang J, Kapli P, Pavlidis P, Stamatakis A. A general species delimitation method with applications to phylogenetic placements. Bioinformatics. 2013;29:2869–76. 10.1093/bioinformatics/btt499 23990417 PMC3810850

[31] Balaban M, Sarmashghi S, Mirarab S. APPLES: scalable distance-based phylogenetic placement with or without alignments. Syst Biol. 2020;69:566–78. 10.1093/sysbio/syz063 31545363 PMC7164367

[32] Letunic I, Bork P. Interactive Tree Of Life (iTOL) v5: an online tool for phylogenetic tree display and annotation. Nucleic Acids Res. 2021;49(W1):W293–6. 10.1093/nar/gkab301 33885785 PMC8265157

[33] RStudio Team. RStudio: integrated development for R [cited 2022 Jan 12]. https://www.rstudio.com

[34] Sergeant ESG. Epitools epidemiological calculators. Ausvet. 2018 [cited 2022 Jan 14]. http://epitools.ausvet.com.au

[35] Körner S, Makert GR, Ulbert S, Pfeffer M, Mertens-Scholz K. The prevalence of *Coxiella burnetii* in hard ticks in Europe and their role in Q fever transmission revisited—a systematic review. Front Vet Sci. 2021;8:655715. 10.3389/fvets.2021.655715 33981744 PMC8109271

[36] Gürtler L, Bauerfeind U, Blümel J, Burger R, Drosten C, Gröner A, et al. *Coxiella burnetii—*pathogenic agent of Q (query) fever. Transfus Med Hemother. 2014;41:60–72. 10.1159/000357107 24659949 PMC3949614

[37] Mediannikov O, Matsumoto K, Samoylenko I, Drancourt M, Roux V, Rydkina E, et al. *Rickettsia raoultii* sp. nov., a spotted fever group rickettsia associated with *Dermacentor* ticks in Europe and Russia. Int J Syst Evol Microbiol. 2008;58:1635–9. 10.1099/ijs.0.64952-0 18599708

[38] Parola P, Rovery C, Rolain JM, Brouqui P, Davoust B, Raoult D. *Rickettsia slovaca* and *R. raoultii* in tick-borne Rickettsioses. Emerg Infect Dis. 2009;15:1105–8. 10.3201/eid1507.081449 19624931 PMC2744242

[39] Li H, Zhang PH, Huang Y, Du J, Cui N, Yang ZD, et al. Isolation and identification of *Rickettsia raoultii* in human cases: a surveillance study in 3 medical centers in China. Clin Infect Dis. 2018;66:1109–15. 10.1093/cid/cix917 29069294

[40] Jia N, Zheng YC, Ma L, Huo QB, Ni XB, Jiang BG, et al. Human infections with *Rickettsia raoultii*, China. Emerg Infect Dis. 2014;20:866–8. 10.3201/eid2005.130995 24750663 PMC4012798

[41] Mendoza-Roldan JA, Colella V, Lia RP, Nguyen VL, Barros-Battesti DM, Iatta R, et al. *Borrelia burgdorferi* (sensu lato) in ectoparasites and reptiles in southern Italy. Parasit Vectors. 2019;12:35. 10.1186/s13071-019-3286-1 30646928 PMC6332633

[42] Sgroi G, Iatta R, Veneziano V, Bezerra-Santos MA, Lesiczka P, Hrazdilová K, et al. Molecular survey on tick-borne pathogens and *Leishmania infantum* in red foxes (*Vulpes vulpes*) from southern Italy. Ticks Tick Borne Dis. 2021;12:101669. 10.1016/j.ttbdis.2021.101669 33578255

[43] Collares-Pereira M, Couceiro S, Franca I, Kurtenbach K, Schäfer SM, Vitorino L, et al. First isolation of *Borrelia lusitaniae* from a human patient. J Clin Microbiol. 2004;42:1316–8. 10.1128/JCM.42.3.1316-1318.2004 15004107 PMC356816

[44] Aguero-Rosenfeld ME, Wormser GP. Lyme disease: diagnostic issues and controversies. Expert Rev Mol Diagn. 2015;15:1–4. 10.1586/14737159.2015.989837 25482091

[45] Budachetri K, Kumar D, Crispell G, Beck C, Dasch G, Karim S. The tick endosymbiont *Candidatus* Midichloria mitochondrii and selenoproteins are essential for the growth of *Rickettsia parkeri* in the Gulf Coast tick vector. Microbiome. 2018;6:141. 10.1186/s40168-018-0524-2 30103809 PMC6090677

[46] Cafiso A, Bazzocchi C, De Marco L, Opara MN, Sassera D, Plantard O. Molecular screening for *Midichloria* in hard and soft ticks reveals variable prevalence levels and bacterial loads in different tick species. Ticks Tick Borne Dis. 2016;7:1186–92. 10.1016/j.ttbdis.2016.07.017 27521265

[47] Buysse M, Duron O. Multi-locus phylogenetics of the *Midichloria* endosymbionts reveals variable specificity of association with ticks. Parasitology. 2018;145:1969–78. 10.1017/S0031182018000793 29779502

[48] Matsumoto K, Izri A, Dumon H, Raoult D, Parola P. First detection of *Wolbachia* spp., including a new genotype, in sand flies collected in Marseille, France. J Med Entomol. 2008;45:466–9. 10.1093/jmedent/45.3.466 18533441

[49] Fraihi W, Fares W, Perrin P, Dorkeld F, Sereno D, Barhoumi W, et al. An integrated overview of the midgut bacterial flora composition of *Phlebotomus perniciosus*, a vector of zoonotic visceral leishmaniasis in the Western Mediterranean Basin. PLoS Negl Trop Dis. 2017;11:e0005484. 10.1371/journal.pntd.0005484 28355207 PMC5386300

[50] Dantas-Torres F, Otranto D. Best practices for preventing vector-borne diseases in dogs and humans. Trends Parasitol. 2016;32:43–55. 10.1016/j.pt.2015.09.004 26507152

