# Supplementary material for: Detection of Endosymbiont Candidatus Midichloria mitochondrii and Tickborne Pathogens in Humans Exposed to Tick Bites, Italy
Source: Emerg Infect Dis. 2022 Sep;28(9):1824–32. doi: 10.3201/eid2809.220329 (PMC9423927; doi:10.3201/eid2809.220329)
Supplement: Appendix — Additional information on detection of endosymbiont Candidatus Midichloria mitochondrii and tickborne pathogens in humans exposed to tick bites, Italy. [file 22-0329-Techapp-s1.pdf]

Article DOI: https://doi.org/10.3201/eid2809.220329

Detection of Endosymbiont *Candidatus* Midichloria mitochondrii and Tickborne Pathogens in Humans Exposed to Tick Bites, Italy

Appendix

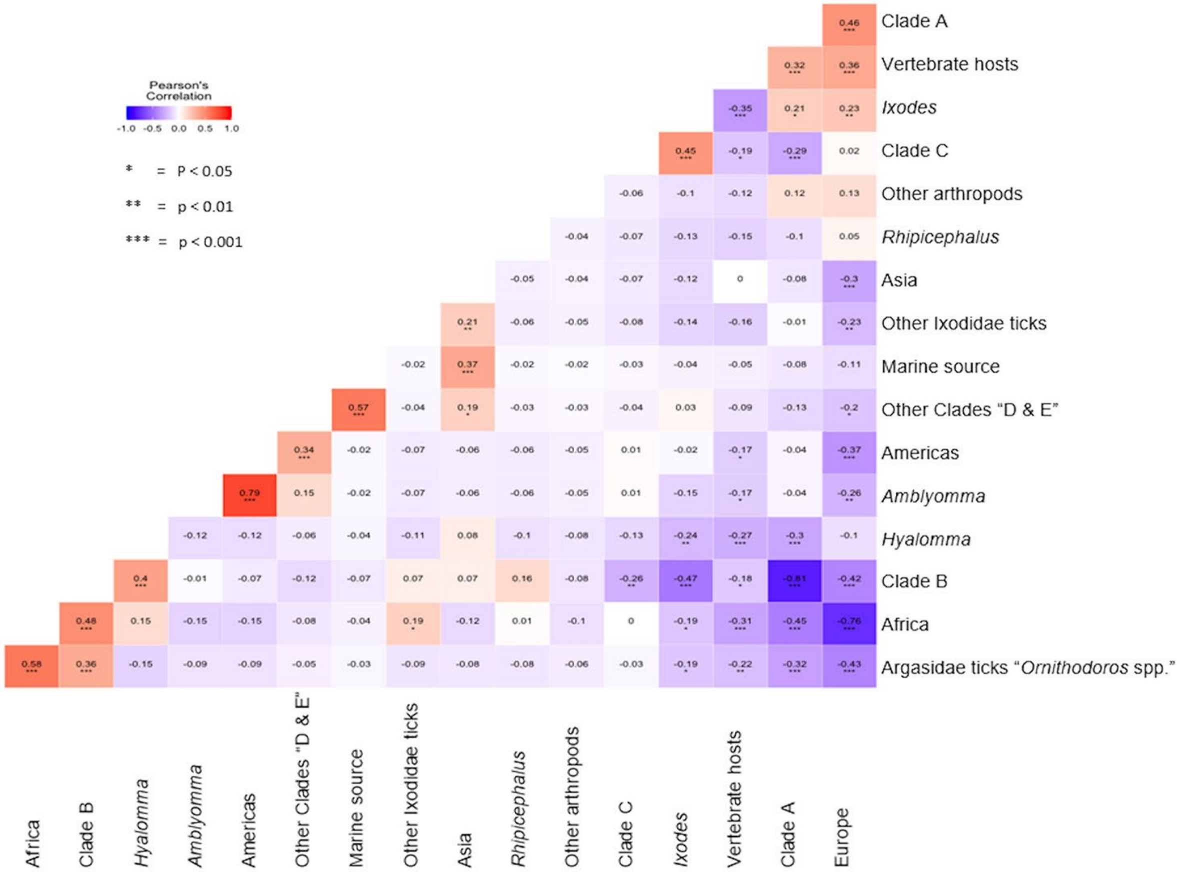

Appendix Figure. Pearson correlation matrix showing the association of *Candidatus* Midichloria mitochondrii clades and tickborne pathogens in humans exposed to tick bites, Italy, 2021. The matrix different vertebrate hosts, tick species, and geographic origin.
